# Supplementary material for: Cytokine Profiles Differentiate Symptomatic from Asymptomatic PTSD in Service Members and Veterans with Chronic Traumatic Brain Injury
Source: Biomedicines. 2022 Dec 19;10(12):3289. doi: 10.3390/biomedicines10123289 (PMC9775258; doi:10.3390/biomedicines10123289)
Supplement: Supplementary file 1 [file biomedicines-10-03289-s001.zip › biomedicines-2061674-supplementary.pdf]

Supplemental Table S1. Group differences in cytokine concentrations

Biomarker (pg/mL)	PTSD symptomatic, N = 62[1]	PTSD asymptomatic, N = 61[1]	p-value[2]	Cohen's d
CRP	[illegible],187,726.228 (589351.477, 2804246.18[illegible]	[illegible]905,713.190 (553516.757, 1940287.221)	0.093	0.44
IL1RA	197.514 (127.337, 290.581)	161.455 (124.913, 283.968)	0.427	0.26
VEGF	40.574 (27.980, 68.262)	45.579 (29.332, 68.159)	0.614	0.01
IL6	0.206 (0.149, 0.298)	0.134 (0.095, 0.227)	< 0.001*	0.50
IL8	2.410 (1.618, 3.284)	1.592 (1.235, 2.129)	< 0.001*	0.84
IL10	0.120 (0.084, 0.181)	0.096 (0.062, 0.134)	0.026*	0.55
TNFα	0.784 (0.354, 1.037)	0.265 (0.223, 0.358)	< 0.001*	1.34
ptau	1.993 (1.555, 2.800)	2.063 (1.504, 3.153)	0.398	0.35

[1] Data are represented as median (IQR)

[2] Mann Whitney U test, $p^* < 0.05$

Abbreviations: CRP, C-reactive protein; IL1RA, interleukin 1 receptor agonist; VEGF, vascular endothelial growth factor; IL6, interleukin 6; IL8, interleukin 8; IL10, interleukin 10; TNFα, tumor necrosis factor alpha; ptau, phosphorylated tau
